# Simple Patch Closure for Perforated Peptic Ulcer in Children Followed by Helicobacter Pylori Eradication

**DOI:** 10.12669/pjms.303.4705

**Published:** 2014

**Authors:** Turan Yildiz, Huri Tilla Ilce, Canan Ceran, Zekeriya Ilce

**Affiliations:** 1 Turan Yildiz, Department of Pediatric Surgery, Sakarya University Medical Schools, Sakarya, Turkey.; 2 Huri Tilla Ilce, Department of Nucleer Medicine, Sakarya University Medical Schools, Sakarya, Turkey.; 3 Canan Ceran, Department of Pediatric Surgery,Inonu University Medical Schools, Malatya, Turkey.; 4 Zekeriya Ilce,Department of Pediatric Surgery, Sakarya University Medical Schools, Sakarya, Turkey.

**Keywords:** Children, Peptic Ulcer, Perforation, Simple Closure

## Abstract

***Objective:*** Peptic ulcer disease in children is rare. Therefore, the diagnosis can be missed until complications such as perforation or hemorrhage occur. Few reports have investigated the procedures and outcomes of children who have undergone operations for perforated duodenal ulcers. We report our experience with the modified Graham technique for perforated duodenal ulcers in nine children and review the literature.

***Methods*** *:* The records of patients operated on for a perforated duodenal ulcer in the last 8 years in two pediatric surgery centers were evaluated retrospectively. Patient demographics, symptoms, time to admission to hospital, operative findings, and postoperative clinical course were evaluated.

***Results*** *:* Nine children (mean age 13.2 years, range 6–170 years) were included. All patients were admitted in the first six hours after their abdominal pain started. In three patients, there was free air on plain x-rays, while the x-rays were normal in six. All perforations were located on the anterior surface of the first part of the duodenum and repaired with primary suturing and Graham patch omentoplasty. The recovery was uneventful in all patients. In five patients, urea breath tests were performed postoperatively for Helicobacter Pylori, and the results were positive. All patients underwent triple therapy with lansoprazole, amoxicillin, and clarithromycin. The mean follow-up time was 58 (range 3–94) months.

***Conclusions*** *:* Peptic ulcer perforation should be suspected in children who have acute abdominal pain and peritoneal signs, especially when their suffering is intense. The simple patch repair and postoperative triple therapy for *Helicobacter Pylori* are safe and satisfactory for treating peptic ulcer perforation in children.

## INTRODUCTION

In adults, histamine-2 receptor blockers, proton pump inhibitors and treatment of *Helicobacter pylori* have already replaced the role of elective surgery in peptic ulcer disease (PUD).^1^ Current treatment modality has also decreased the rate of elective surgery, but emergent surgical conditions such as peptic ulcer perforation ( PUP ), bleeding or obstruction have not been decreased.^2^ However, effects of improvement in the management in children are not apparent as in adults because the literature on the subject is uncommon. 

Various surgical procedures have been advocated for patients with PUP, ranging from simple closure with an omental patch, vagotomy, to gastrectomy. Omental patch (Graham patch) closure of perforated duodenal ulcers was first described in 1929 by Cellen-Jones and later by Graham in 1937.^3^

 Most of the literature on PUP is on adults; PUP has been investigated less frequently in children.^3^ This study reports our experience with the Graham patch repair for perforated duodenal ulcers in nine children and reviews the literature.

## METHODS

The records of patients operated on for PUP in the last 8 years were reviewed retrospectively. The patients’ age at diagnosis, sex, family history of peptic ulcer disease, history of chronic abdominal pain, coexisting clinical events, clinical findings, time between symptom onset and admission to hospital, operative findings and postoperative outcome were evaluated (Table-I). PUP repair using the primary suturing and a Graham patch omentoplasty technique, as described previously, was performed on all patients under general anesthesia.^4^

## RESULTS

**Fig.1:**
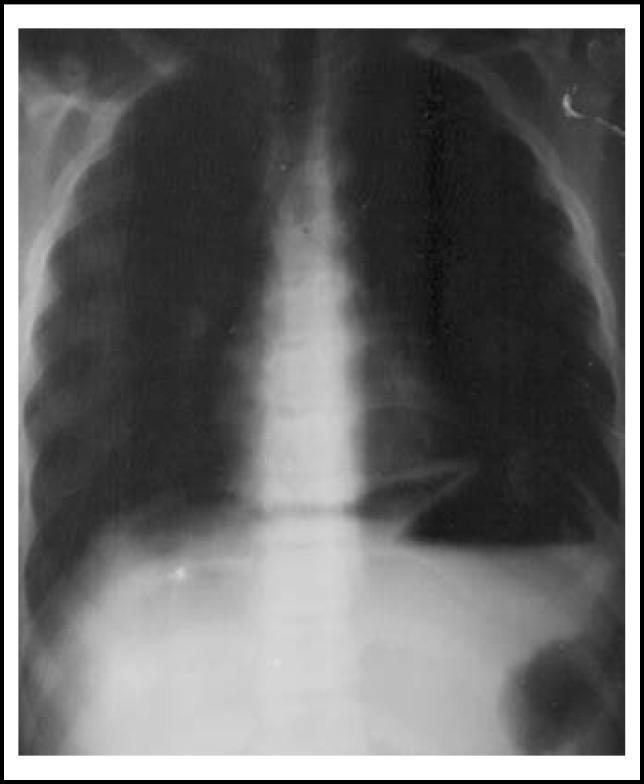
Subdiaphragmatic free air in patient

Nine children age 6–17 years (mean age 13.2 years) were included in the study, one female and eight males. All patients were admitted in the first six hours after their abdominal pain started. In three patients, plain x-rays revealed subdiaphragmatic free air (Fig.1), while the x-rays were normal in six patients. All perforations were located on the anterior surface of the first part of the duodenum and repaired with primary suturing and a Graham patch omentoplasty. There were no large perforations; all were less than 0.8 cm in diameter. Nasogastric tube drainage was used for 3 days postoperatively. One patient who had no free air on x-ray was thought to have appendicitis, so a laparotomy was performed with a right lower transvers incision. The appendix was normal and there was fluid containing food in the abdomen. The first incision was closed and an upper midline incision made for repair of duodenal perforation. In another patient, the diagnosis was unclear, so a diagnostic laparoscopy was performed.

To repair the perforation, the abdomen was entered with a midline incision; the perforation was exposed and surrounding tissues and spaces irrigated. Sutures were placed across the perforations. An omental patch was mobilized on a vascular pedicle, placed over the perforation, and secured with sutures. The abdomen was then closed.

Oral full feeding/intake was achieved on 5^th^ postoperative day. The recovery period was uneventful in all patients. In five patients, urea breath tests were performed; all results were positive. All patients underwent triple therapy for *Helicobacter pylori* eradication (Helipak; Fako Ilaclari A.Ş. Levent – Istanbul, Turkey) comprising lansoprazole 1 mg/kg/day, amoxicillin 50 mg/kg/day and clarithromycin 15 mg/kg/day for 2 weeks. The lansoprazole treatment was continued for 6 months. The mean follow-up time was 58 (range 3–94) months. One patient complained of abdominal pain 2 years after therapy and was administered a repeat course of medical therapy.

## DISCUSSION

Peptic ulcer disease (PUD) is comparatively uncommon condition in childhood; however, more often application of endoscopy in children led the PUD diagnosis more frequently.^1^ Introduction of histamine 2 (H2) receptor blockers, proton pump inhibitors, and treatment for *H*. *pylori* in medical treatment had diminished the need of elective surgery, but emergency complications surgery has not been reduced, so emergency operation could be required.^3^ PUP occurs in nearly 10% of adults^5^ and 12.5% of children^6^ with PUD.

 PUP is usually assessed in two groups, primary and secondary PUD. Primer PUD is not associated with another disease but most cases of primary peptic ulcers are associated with *H*. *pylori* infection. Severe stresses, such as with systemic illnesses, burns, head trauma, and Crohn’s disease^7^ and induced by ingestion of non-steroidal anti-inflammatory drugs can be accompanied by Secondary ulceration.^8^ Our patients had no underlying diseases. All of the patients had primary peptic ulcers.

PUP is one of the reasons in acute abdomen. But this clinical condution may be masked with presence of other illness. Chronic or recurrent symptoms in primary peptic ulcer disease could occur. Most children present with episodic abdominal pain. Additionally, that should be associated with vomiting and nocturnal awakening. Up to 90% of children with ulcers are diagnosed with endoscopy that they have abdominal pain.^9^ The children in this study did not have any chronic or recurrent complains in their medical histories and endoscopy examination was not performed in any child. All children presented within 6 h of their acute, sudden-onset abdominal pain.

If one is suspicious, PUP diagnosis can be easy. Sudden onset abdominal pain and generalize peritonitis with board like abdomin are pathognomonic in PUP. Free air under the diaphragm can be seen on plain abdomen radiography in about 90% of patients. Other investigations are most often not required in an emergency situation.^10^^,^^11^ If the diagnosis is not confirmed, additional investigations would be required. Computed tomography (CT) is more sensitive than plain radiographs for detecting free air in intraabdominal; CT will typically demonstrate perforations and can be used to assess the presence of free versus contained perforations.^10^ In addition, laparoscopy can be used for diagnosis and treatment.^4^ In three of the nine children, we established a diagnosis of PUP from plain x-rays of the abdomen. In six of our patients, there was no gas on the plain x-rays. We did not use CT in any patient and the final diagnosis of six cases were established on laparotomy and laparoscopy. In one case, the incision was based on a preliminary diagnosis of appendicitis, but appendix was normal. So incision of appendicitis was closed and replaced. In another case, a diagnostic laparoscopy was performed, but open surgery was performed for treatment, due to lack of experience with laparoscopic surgery and technical reasons. But from now on we are going to perform laparoscopic surgery for PUP.

The surgical treatment of PUP depends upon the severity of the disease and extent of perforation. Most small perforations can be treated by simple closure with an omental patch. This may be performed by open or laparoscopic techniques. In complex or giant perforated duodenal ulcers, definitive procedure like vagotomy or gastric resections may be required. In the era before the *H*. *pylori* treatment studies demonstrated better long-term outcomes with definitive operations for the ulcer perforations.^4^^,^^12^

Better outcomes in closure of the ulcer perforation was obtained with an understanding of the importance of H. pylori in the pathogenesis of duodenal ulcer.^13^ Incidence of H. Pylori in patients with chronic peptic ulcers is similar in most studies.^6^ Therefore, the postoperative follow-up of ulcer perforation should include that antibacterial and antisecretory therapy should be administered to infected patients to eradicate H. Pylori. Ulcer healing and eradication of H. Pylori is recommended with endoscopy after six weeks postoperatively.^11^ Relapse of duodenal ulcer following simple omental patch in PUP rate up to 42 % . Bose *et al*. reported that *H*. *pylori* infection causes duodenal ulcer relapse following simple closure of PUP.^13^ The treatment of *H*. *pylori* infection significantly reduces duodenal ulcer relapse. Therefore anti-*H*. *pylori* therapy should be administered to all patients with perforated duodenal ulcers who are positive for *H*. *pylori* infection after simple closure.^14^ In this study, we did not apply invasive endoscopy for diagnosis of h pylori. But instead noninvasive urea breath teste was used in most patients with PUP. We also applied, urea breath teste irrespective of the fact whether, the triple therapy for h.pylori was used or not in PUP patients.

**Table-I:** Patient characteristics

	*Case 1*	*Case 2*	*Case 3*	*Case 4*	*Case 5*	*Case 6*	*Case 7*	*Case 8*	*Case 9*
Age and Sex	13 Y/M	16 Y/F	14 Y/M	15 Y/M	6 Y/M	9 Y/M	17Y/M	14 Y/M	15Y/M
Duration of abdominal pain (hours)	3 h	5 h	3 h	5 h	2 h	5 h	5 h	4 h	5 h
History of chronic abdominal pain	-	+	+	+	+	+	+	+	+
Family history of PUD	-	+	+	+	+	+	+	-	-
X-Ray (Sub diaphragmatic free air)	+	+	-	+	-	-	-	-	-
Urea breath test	HP+	HP+	HP+	HP +	Not done	HP+	Not done	Not done	Not done
Follow up (months)	94	39	59	61	80	86	3	24	76
Leucosyt	11000	11000	12100	19000	18500	15000	9000	12000	10000
Duration of drainage (day)	3	3	3	3	2	3	2	3	3
Oral full feading (day)	5	5	5	5	4	5	4	4	4

We applied a Graham patch in our patients based on adult reports. Most PUP patients have very high rates of *H*. *pylori* infection and the rate of recurrence is high in those with untreated* H*. *pylori *infection. Therefore, postoperative medical treatment of *H*. *pylori* is mandatory. 

## CONCLUSION

Peptic ulcer perforation (PUP) in children is an uncommon cause of acute abdomin. PUP should be considered in patients with sudden abdominal pain, especially recurrent history of abdominal pain. In these patients, the peptic ulcer disease is usually associated with *H*. *pylori* infection. The non-invasive urea breath test should be performed to confirm the presence of *H*. *pylori*. A Graham patch is the preferred treatment of PUP in childhood, and this method is simple and safe. In addition, the triple therapy should be given for *H*. *pylori* postoperatively.

## Authors Contribution:

**TY** conceived, designed and did statistical analysis & editing of manuscript.

**HTI, CC** did data collection and manuscript writing.

**ZI** did review and final approval of manuscript.

**TY** takes the responsibility and is accountable for all aspects of the work in ensuring that questions related to the accuracy or integrity of any part of the work are appropriately investigated and resolved.
